# Robust Soil Water Potential Sensor to Optimize Irrigation in Agriculture

**DOI:** 10.3390/s22124465

**Published:** 2022-06-13

**Authors:** David Menne, Christof Hübner, Dennis Trebbels, Norbert Willenbacher

**Affiliations:** 1 Institute for Mechanical Process Engineering and Mechanics, Karlsruhe Institute of Technology, 76131 Karlsruhe, Germany; norbert.willenbacher@kit.edu; 2 TRUEBNER GmbH, 67435 Neustadt an der Weinstraße, Germany; c.huebner@truebner.de (C.H.); d.trebbels@truebner.de (D.T.)

**Keywords:** porous matrix sensor, porous ceramic, capillary suspensions, matric potential, soil water potential sensor, time-domain transmission, dielectric measurement

## Abstract

Extreme weather phenomena are on the rise due to ongoing climate change. Therefore, the need for irrigation in agriculture will increase, although it is already the largest consumer of water, a valuable resource. Soil moisture sensors can help to use water efficiently and economically. For this reason, we have recently presented a novel soil moisture sensor with a high sensitivity and broad measuring range. This device does not measure the moisture in the soil but the water available to plants, i.e., the soil water potential (SWP). The sensor consists of two highly porous (>69%) ceramic discs with a broad pore size distribution (0.5 to 200 μm) and a new circuit board system using a transmission line within a time-domain transmission (TDT) circuit. This detects the change in the dielectric response of the ceramic discs with changing water uptake. To prove the concept, a large number of field tests were carried out and comparisons were made with commercial soil water potential sensors. The experiments confirm that the sensor signal is correlated to the soil water potential irrespective of soil composition and is thus suitable for the optimization of irrigation systems.

## 1. Introduction

Global climate change continues to progress. In the year 2020, global temperatures tied record highs [1]. For this reason too, even more urgent attention must be paid to the waste of water and the associated consequences regarding the water supply of mankind. Agriculture is responsible for >70% of the water consumption worldwide [2,3]. At the same time, water losses of >50% are state-of-the-art in this sector. Accordingly, this is where the leverage for sustainable and efficient water use is the greatest and of crucial importance for mankind [4,5].

The sustainable use of the worldwide available water resources is only possible using modern monitoring and control techniques, especially due to increasing conflicts of objectives, such as between environmental protection and crop production [6]: on the one hand, the washing out of nutrients is to be prevented by a sustainable water supply; on the other hand, ever-increasing quality demands require increasingly intensive cultivation [7]. The technology of irrigation systems has already reached a high level. However, the associated measurement systems for determining soil moisture in agriculture are limited [8,9,10].

To determine the water content of a soil, one possibility is to use the water retention curve. The water retention curve relates the volumetric water content to the soil water potential Ψ_t_, (SWP), i.e., the specific potential energy of the water in the soil. The SWP can be expressed as the sum of normalized forces acting on the water in the soil (see Equation (1)). It includes the gravitational-(Ψ_g_), matric-(capillary and adsorptive, Ψ_m_), osmotic- (Ψ_o_), and hydrostatic potential (Ψ_h_). All the forces emanating from the soil matrix are summarized in the matric potential [5].

Ψ_t_ = Ψ_g_ + Ψ_m_ + Ψ_o_ + Ψ_h_ (1)

The SWP does not indicate the volumetric water content but the fraction of water available to plants. Therefore, soil water potential sensors are not only essential for the most efficient use of water in agriculture but for Earth system models [11]. The most common sensors for measuring the soil water potential are piezometers, tensiometers, heat dissipation sensors, thermocouple psychrometers, and dielectric sensors [5]. The advantages and disadvantages of the individual devices are summarized in ref. [12]: classic tensiometers have a limited measurement range and require frequent maintenance; heat dissipation sensors have a high power consumption and a slow reaction time. In contrast, dielectric sensors are inexpensive, require little maintenance, and have low power consumption [12]. Only a few sensors described in the literature are commercially available, and the market is dominated by a small number of devices (see Table 1) [8,9,10]. For further information on commercially available sensor systems, see ref. [10]. In addition to the mentioned commercially available sensors, Whalley [13,14,15] developed a porous matrix sensor that shows a measuring range from 500 hPa to 3000 hPa. Recently, a few new sensors have been introduced in the literature, which could market-launch in the near future [16,17,18,19,20,21,22,23,24,25].

In conclusion, a sensor is still needed, which offers a low-cost alternative, covers the whole measuring range, from very dry soils often addressed in soil science to very wet soils relevant in agriculture, and offers good performance in saline, vertic, and stony soils [8].

In our sensor principle, the dielectric constant of a porous ceramic disk is determined with a high-frequency measurement. The porous ceramic disk is in hydraulic equilibrium with the surrounding soil [33]. In this way, water is transported into or out of the ceramic depending on the water potential. The water content in the ceramic is determined via the resonant frequency of the electrical resonant circuit. The ceramic has a particularly high pore volume and a wide pore size distribution in order to obtain a large measuring effect. By measuring the dielectric permittivity of the porous ceramic disk, the water potential is determined. In this way, the water potential of the soil can be calculated using correlation functions for standard soils [34].

The main component of our dielectric sensor is two highly porous ceramic discs. The ceramic has pore sizes between 0.5 µm and 200 µm, with an open porosity of well over 60% and a sufficiently high mechanical strength. This is enabled by using so-called capillary suspensions as a precursor for the production of highly porous ceramics. Capillary suspensions are ternary solid/fluid/fluid systems consisting of microscopic particles suspended in a main liquid and a small proportion of an immiscible secondary liquid. The capillary forces inferred by the secondary liquid trigger the formation of a sample spanning particle network. The particle network, stabilized by the strong capillary forces, remains largely intact even after the main liquid has been removed so that highly porous green bodies can be formed. These green bodies are then sintered to produce open-pore ceramic parts. With this concept, the pore shape, pore size distribution, porosity, and mechanical strength can be adjusted over a wide range by varying the particle type, size, and shape [35,36,37].

## 2. Materials and Methods

### 2.1. Raw Material

The raw powders used to fabricate the ceramic discs were commercial-grade brown kieselguhr with a volume-based particle diameter x_50,3_ of 12.0 µm and a density of 2.57 g/cm^3^ and white kieselguhr with an x_50,3_ of 33.5 µm and a density of 2.24 g/cm^3^ (VWR International GmbH, Karlsruhe, Germany). The initial particle size distribution of the employed powders and soils was determined according to DIN EN ISO 8130-13 using a commercial Fraunhofer diffraction device (Helos H0309; Sympatec, Clausthal-Zellerfeld, Germany) equipped with an ultrasonic wet dispersing unit (Quixel, Sympatec) and sieve analysis. The bulk phase used to fabricate the ceramic discs was odorless mineral spirits, (Sigma-Aldrich, Karlsruhe, Germany) with a relative density of 0.752 g/cm^3^, and the secondary phase was a 50 vol.% solution of D(+)-sucrose (Carl Roth, Karlsruhe, Germany) in distilled water prepared at room temperature. Further details on the fabrication process based on capillary suspensions can be found in ref. [34] and on the general manufacturing of capillary suspensions in refs. [36,38].

**Figure 1:**
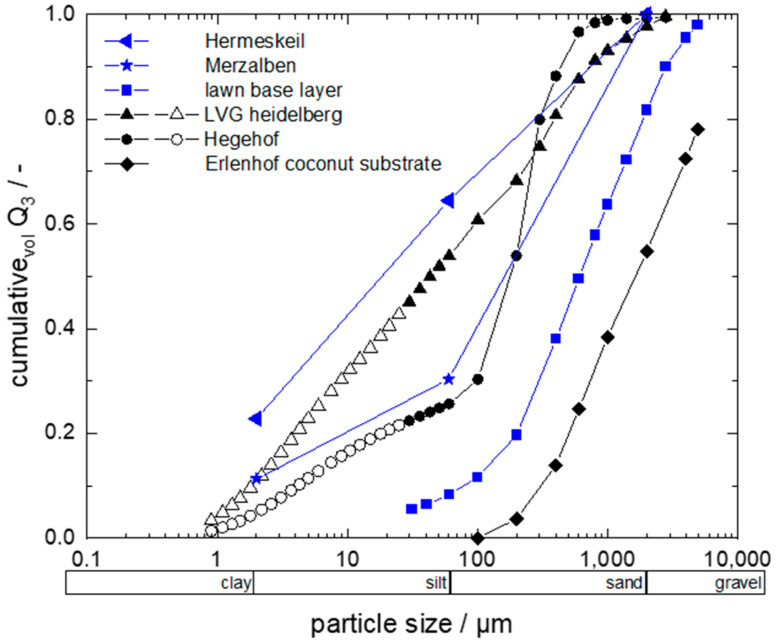
Particle size analysis of the used soils. Cumulative distribution Q_3_ was determined through sieve analysis (open symbols) and through Fraunhofer diffraction (filled symbols).

The cumulative particle size distributions of the model soils are shown in Figure 1. Data shown in blue in the diagram were used for calibration of the sensor. The soil types were a soil at a measuring station of Landesforsten Rheinland-Pfalz forestry administration in Hermeskeil (Rhineland Palatinate High Forest, Germany) consisting of 22.8% clay, 41.6% silt, and 35.6% sand, soil at State Horticultural College and Research Institute (LVG; Heidelberg, Germany) with an x_50,3_ of approximately 45 µm, soil at a measuring station of Landesforsten Rheinland-Pfalz forestry administration in Merzalben (Rhineland Palatinate High Forest, Germany) consisting of 11.3% clay, 19.0% silt, and 69.7% sand, soil at Hegehof (Hegehof GmbH, Ladenburg, Germany) with an x_50,3_ of approximately 190 µm, a model soil so-called lawn base layer with lavasand (corthum Nordschwarzwald GmbH, Marxzell, Germany) with an x_50,3_ of approximately 700 µm and coconut substrate at Erlenhof (Erdbeerland Funck GbR, Eisenberg, Germany) with an x_50,3_ of approximately 1700 µm.

### 2.2. Ceramic and Sensor Characterization

The open porosity of the ceramic discs was determined using Archimedes’ principle according to DIN EN 993-1 (calculation of Archimedes density) and DIN EN 993-18 (technical implementation). Compressive mechanical strength was measured following DIN51104. Based on image processing using line intercept count method of crosscut images obtained from scanning electron microscopy in backscattering mode (SEM-BSE), pore size distributions were calculated as described in refs. [36,38].

Measurements with just the circuit board in air and in water were used to determine the maximum and minimum measured value of each sensor.

For air drying experiments, the porous ceramics were infiltrated with water for 1 min under atmospheric pressure and room temperature, then set to drying at ambient conditions.

Commercially available soil sensors used in this work were: the SMT100 (TRUEBNER GmbH, Neustadt, Germany), measuring the soil water content using a transmission line within a time domain transmission (TDT) circuit; the TEROS 21 Gen.1, formerly MPS-6 (METER Group, Inc., Munich, Germany formerly Decagon Devices, Inc., USA), a matric potential sensor using an impedance of 70 MHz signal in equivalent porous medium [10]; and the T8 tensiometer (METER Group, Inc., Munich, Germany formerly UMS GmbH, Munich, Germany), a matric potential sensor measuring the tension of water directly at a pressure transducer [10].

## 3. Results

### 3.1. Ceramic and Sensor Properties

We characterized our white and brown ceramics, as well as the ceramics used in the TEROS 21, regarding their open porosity ε_open_, mechanical compressive strength σ_c_, and pore size distribution.

The dimensions, characteristic pore size distribution data, mechanical strength, and porosity of the discs made from the brown and white kieselguhr, as well as those used in the TEROS 21 sensor, are summarized in Table 2.

**Figure 2:**
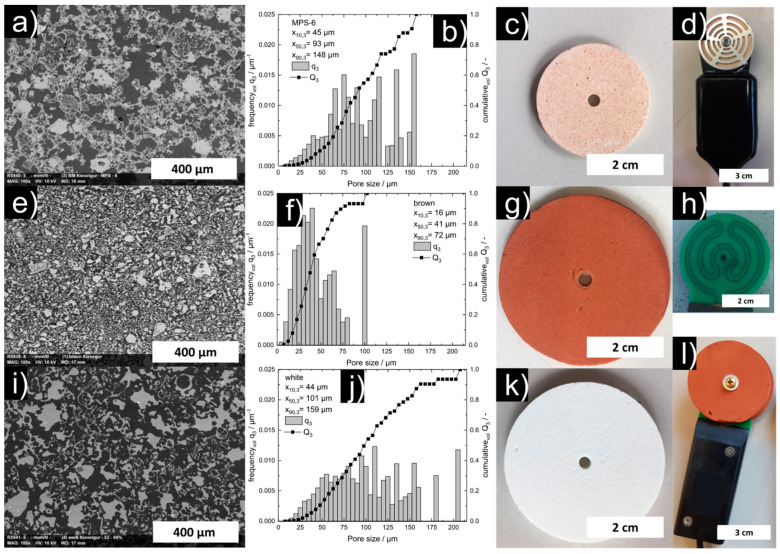
Comparison of the TEROS 21/MPS−6−(**top**), the brown (**middle**), and white ceramics (**bottom**): SEM images of polished surfaces of sintered parts (cavities in black and ceramic material in white) (**a**,**e**,**i**), pore size distribution q_3_ (x_pore_) and Q_3_ (x_pore_) (**b**,**f**,**j**), top view of the ceramics (**c**,**g**,**k**), top view of the sensor (**d**,**l**), and the electrode of the TDT sensor (**h**).

Corresponding SEM images, particle size distribution data, and photographs of the sensors are shown in Figure 2.

Our sensor setup uses a transmission line within a TDT circuit. The propagation velocity of the electrical pulses along this line depends on the capacitance between the electrodes [39]. To confine the electric field to the ceramic disc, the geometric circuit dimensions were specially designed. The transmission line is embedded in a multilayer printed circuit board and forms a ring oscillator with the accompanying electronic circuit. In order to achieve a long geometric length on a small area, the transmission line is folded. The longer the line, the higher the measurement resolution. A detailed schematic of the TDT principle is shown in ref. [33]. The oscillating frequency is a few hundred MHz. Because of that, variations in the electrical conductivity of the water in the ceramic disc, e.g., due to varying salinity, only have a minor effect on the measured signal. The oscillating frequency is measured using a microcontroller and provides the raw data as so-called “counts”. Counts are proportional to the oscillation frequency. This means that a higher count value corresponds to a higher frequency and thus a lower moisture in the soil. The stability of the ring oscillator is about one count, and, together with a dynamic range of over 1000 counts, its resolution is 10 bit [34]. TDT is perfectly suited for saline environments due to the high frequency, and it has the same advantage as a time-domain reflectometer (TDR).

### 3.2. Air Drying Experiments: Laboratory

**Figure 3:**
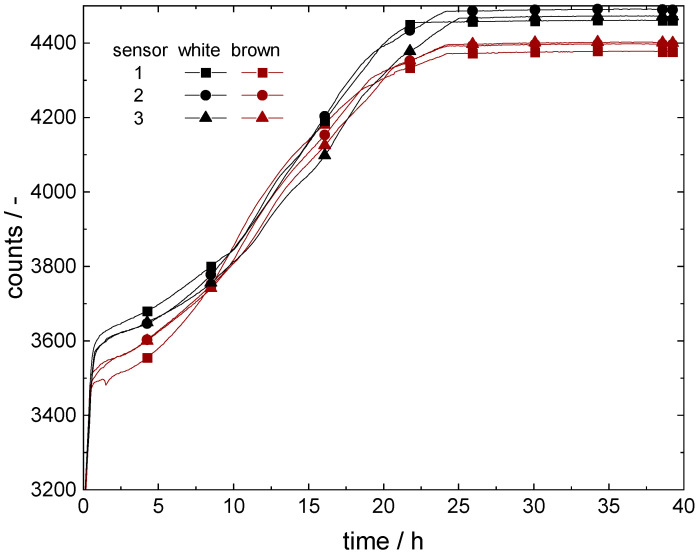
Drying experiment in air at 25 °C: counts are plotted versus time in hours; ceramic drying curves of six different TDT circuit sensors: three sensors with brown and three sensors with white ceramic discs.

The results of the air-drying experiment are shown in Figure 3. Fully infiltrated and submerged in water, the sensors showed count values of approximately 3200 ± 5 counts for both ceramic types. The drying curves of the sensors with both ceramics do not show significant differences, although the ceramics show significant differences in the pore size distribution (cf. Figure 2b,f and ref. [34]). Obviously, the difference in the pore size distribution of the two ceramics is not significant for drying kinetics under atmospheric pressure. In air at a dry state, the sensors mounted with white ceramics showed count values of approximately 4450 ± 5 counts and, for the sensors mounted with brown ceramics, approximately 4400 ± 5 counts.

### 3.3. Soil Moisture Measurement Experiments

To test the electronic layout of the sensor and the different functionality of the two ceramics in the soil and to test the sensor, soil moisture measurement experiments were conducted in soils with different particle size distributions.

The aim of these experiments was to find out the potential of the sensor for optimized irrigation, collect sufficient data to correlate measured counts with soil water potential, and to compare the functionality of our sensor with commercially available sensors.

For the experiments, sensors mounted with brown and white ceramic discs were installed at Hegehof, Erlenhof, LVG, and at a measuring station at Hermeskeil in the Palatinate Forest in cooperation with the Landesforsten Rheinland-Pfalz forestry administration. A dielectric sensor for volumetric water content (SMT100), a tensiometer (T8), and a soil water potential sensor (TEROS 21) were used in this measurement campaign for comparison.

#### 3.3.1. Hegehof

**Figure 4:**
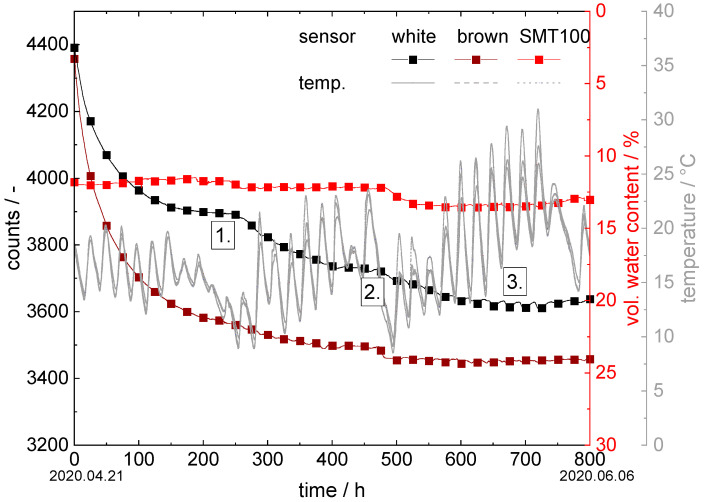
Soil moisture measurement experiment at Hegehof in asparagus field: counts are plotted versus time in hours for our new sensors; vol. water content vs. time for the SMT100.

The sensors were installed in an asparagus bed at a depth of approximately 15 cm under plastic foil. Irrigation took place only through natural precipitation. The measuring period examined was between 21 April 2020 and 6 June 2020. In Figure 4, the variation in the temperature during this is shown. The temperature in the soil ranged between a minimum of 10 °C and a maximum of 30 °C and reproduces the usual temperature fluctuations between day and night. All the sensors exhibited the same temperatures during this trial. Furthermore, Figure 4 shows the corresponding soil moisture measurement values for the sensors with brown and white ceramic discs, as well as for the SMT100.

The curves can be divided into three sections: the first section lasts up to about 250 h, the second to about 475 h, and the last period extends to the end of the measuring period at 800 h. In the first range, the sensors with brown and white ceramic discs form a hydraulic equilibrium at approximately 3900 counts (white) and at approximately 3500 counts (brown). At the end of the first regime and in the transition to the second regime, precipitation took place on the fields. The sudden drop in temperature during the time interval between 200 h and 250 h also indicates an atmospheric low-pressure period. The counts of the sensor with the white ceramic discs, therefore, dropped to 3750 counts and those with the brown ceramic discs to approximately 3700 counts. The sensors reacted here with a strong delay since the precipitation is only slowly absorbed into the asparagus soil wall due to the protective plastic film covering it. In the third period, the process of natural precipitation was repeated. Here, the count values for the sensor with white ceramic discs reached a minimum of 3600 and those for the sensor with brown ceramic discs a minimum of 3450. Both precipitation events were also recorded by the SMT100: the volumetric water content increased from the first to the second regime from 11.5 vol.% to 12.2 vol.% and, finally, to 13.5 vol.% in period three.

Although the SMT100 provides a relatively accurate indication of the increasing moisture in the soil, it is not possible to determine how much soil water is available for the plants.

The difference in the measurement values of the sensors with brown and white ceramic discs can be attributed to the different pore size distributions. The pore size distribution of the brown ceramic is much narrower than that of the white ceramic and has predominantly small pores. As a result, the brown ceramics exhibit a higher capillary effect and, in this case, extract more water from the surrounding soil than the white ceramics.

#### 3.3.2. Erlenhof

For the experiments at the Erlenhof, a total of four sensors were installed in two 50 L pots between 27 April 2020 and 4 August 2020. The pots were filled with coconut substrate and planted with blueberries. The blueberries were regularly irrigated (three times per day) by an automatic irrigation system.

**Figure 5:**
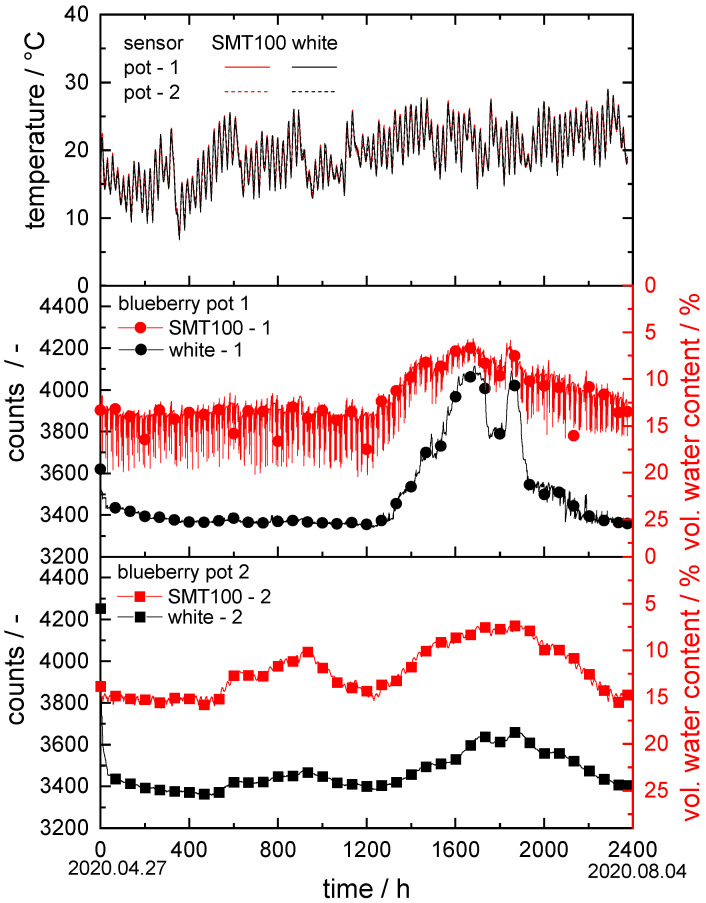
Soil moisture measurement experiment at Erlenhof in two blueberry pots: the upper graph shows temperature vs. time at all sensor positions; in the middle and lower graphs, counts are plotted vs. time in hours for our new sensors; vol. water content vs. time for the SMT100.

In Figure 5, the corresponding temperature is plotted versus the time for all sensors in both blueberry pots. Further, in Figure 5, the corresponding soil moisture measurement curves are shown with counts plotted versus time for the sensors with white ceramic discs and volumetric water content versus time for the SMT100.

The temperature in the substrate of both pots started at approximately 20 °C on April 27 and fluctuated between a minimum of approximately 10 °C and a maximum of approximately 28 °C in the following weeks. The blueberry pots were located outside under a tunnel tent. The usual temperature fluctuations between day and night are reflected equally by all the sensors. The sensors exhibit no significant difference among each other.

In the first blueberry pot, the SMT100 measured a water content of approximately 13.5 vol.% for the first 1200 h. Subsequently, the water content dropped, reaching a minimum of 6.5 vol.% after 1650 h, and then increased again to approximately 13.5 vol.% in the final measurement period. Over the entire measuring range, the data fluctuated in a range of ±6 vol.%. This could be due to the sensor being installed very close to the irrigation unit. This means that each irrigation cycle is registered directly by the sensor, without the water being able to distribute itself evenly in the substrate beforehand. The sensor mounted with white ceramic discs established a hydraulic equilibrium with the surrounding soil within 24 h at approximately 3400 ± 50 counts. After 1200 h, the counts’ values started to rise, reaching a maximum of approximately 4050 ± 50 counts at 1650 h, then dropped to 3800 ± 50 counts for 100 h, and increased again to approximately 4050 ± 50 counts. Finally, the signal steadily dropped back to 3400 ± 50 counts. Like the SMT100, this sensor was installed close to the irrigation valve. This could allow the water to be absorbed by the sensor’s ceramics during irrigation cycles without being evenly distributed in the substrate.

The data collected in the second blueberry pot were similar to those from the first pot. The SMT100 data, however, exhibit only weak fluctuations in a range of ±0.5 vol.%. This is due to the greater distance between the sensors and the irrigation valve. The measuring curve obtained using the sensor with white ceramic discs ran almost parallel to that of the SMT100, underlining the functionality of the new sensor.

Finally, this experiment shows that both the SMT100 and our sensor with white ceramic discs captured the water content in the coconut substrate, a soil that is very coarse, with an x_50,3_ of approximately 1700 µm, satisfactorily. On the other hand, it also shows that the appropriate placement of the sensor in the substrate has a significant influence on the measurement results obtained.

#### 3.3.3. LVG

**Figure 6:**
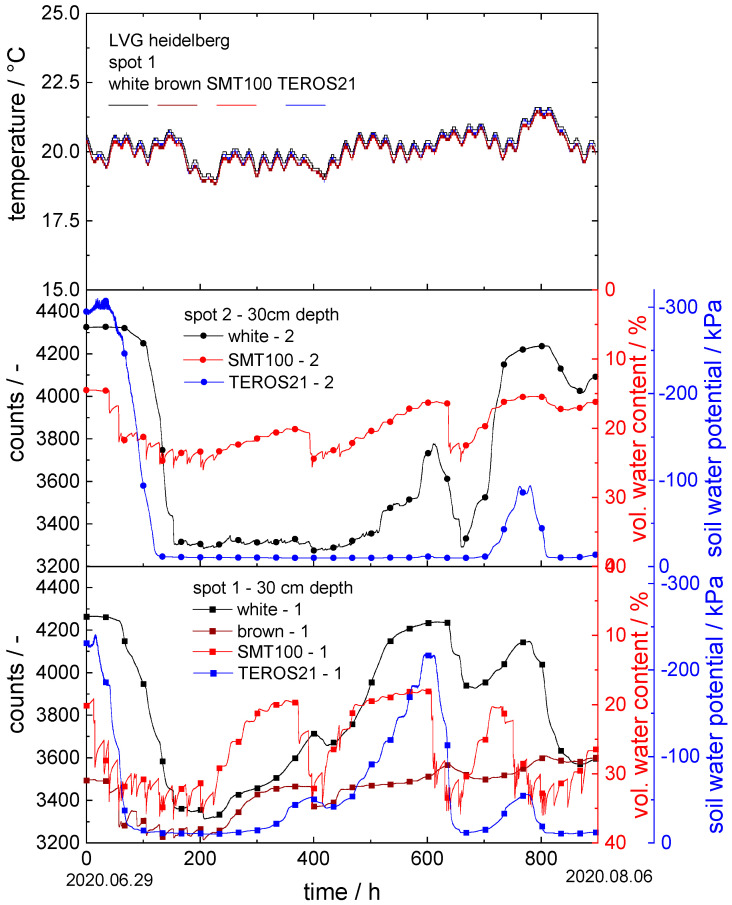
Soil moisture measurement experiment at LVG in tomato research field: the upper graph shows temperature vs. time at all sensor positions; in the middle and lower graphs, counts are plotted vs. time for the new sensors with white and brown ceramic discs; vol. water content vs. time for the SMT100 and soil water potential vs. time for TEROS 21.

**Figure 7:**
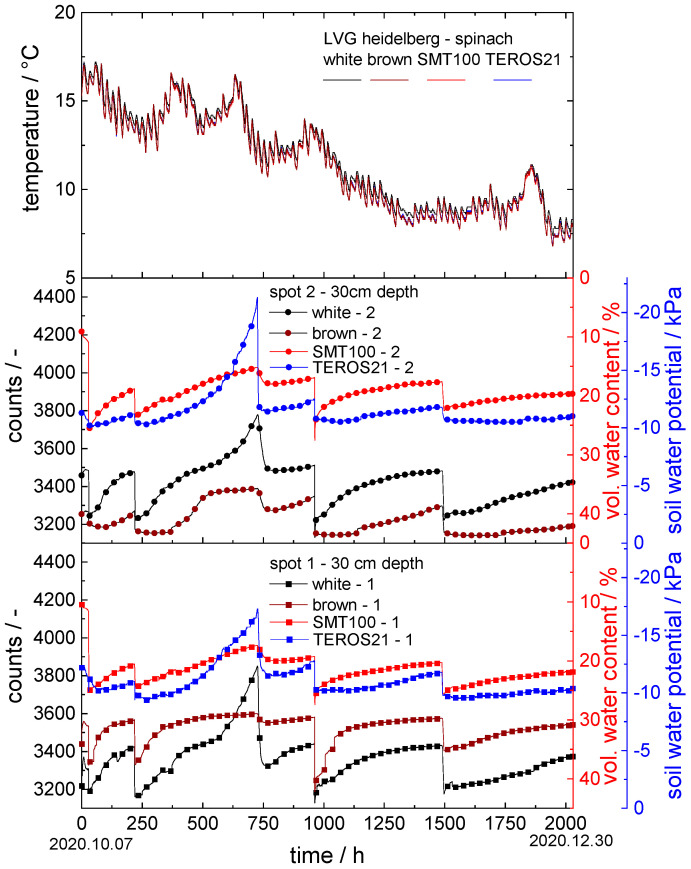
Soil moisture measurement experiment at LVG in spinach research field: the upper graph shows temperature vs. time at all sensor positions; in the middle and lower graphs, counts are plotted vs. time in hours for our new sensors; vol. water content vs. time for the SMT100 and soil water potential vs. time for TEROS 21.

For the experiments at the LVG, a total of seven sensors were installed in the tomato research field (see Figure 6) and eight sensors in the spinach research field (see Figure 7). In the tomato field, the measurements were taken between 29 June 2020 and 6 August 2020 and in the spinach field between 7 October 2020 and 30 December 2020. In both fields, three and four sensors, respectively, were installed at two different locations approximately 1 m apart at a depth of 30 cm. Both fields were regularly irrigated by an automatic irrigation system with a time schedule.

In both Figure 6 and Figure 7, the temperature is plotted versus time for the sensors grouped at spot 1. Furthermore, the corresponding soil moisture data obtained from the sensors with white and brown ceramic discs, the SMT100 and the TEROS 21, are shown.

- Tomato research field:

The temperature in the soil started at approximately 20 °C on 29 June and fluctuated in the range of ±1 °C in the following weeks. The small temperature variations between day and night are reflected equally by all the sensors; the temperature signals detected by the different sensors exhibited no significant difference among each other.

At spot 2, initially a relatively dry area, the first irrigation took place after approximately 50 h, which was registered similarly by all three sensors, but, for the ceramic disc sensor, the respective count drop came with some delay. The soil water potential detected by the TEROS 21 then increased from −300 kPa to −10 kPa within 100 h, the count value obtained by the sensor with white ceramic discs dropped from 4325 counts to 3315 counts in the same time interval, and the water content measured with the SMT100 rose from 14.5 vol.% to 25.6 vol.%. The TEROS 21 then remained at −10 kPa without significant fluctuations until hour 700, then decreased to −90 kPa and finally rose again to −10 kPa, corresponding to the lower measuring limit of the TEROS 21. In contrast, the further drying (after 375, 600, and 800 h) and irrigation (after 400, 650, and 840 h) periods were similarly registered by the SMT100, as well as the ceramic disc sensors.

Since spots 1 and 2 were only about 1 m apart, the drying and watering processes detected by the three sensors were similar to each other. For the SMT100 at spot 1, the irrigation cycles were registered more strongly than at spot 2. For this sensor, for example, the measured water content in the soil even increased to approximately 35 vol.% during the first irrigation period after 100 h. For the sensor with white ceramic discs and the TEROS 21 at spot 1, for example, the drying period after 600 h showed up more pronounced: the sensor with white ceramic discs measured 4235 counts at this spot and the TEROS 21 detected a soil water potential of −215 kPa. In contrast, at spot 2, the TEROS 21 registered no drying phase at all and the sensor with white ceramic discs detected a less pronounced drying phase. In addition, a sensor with brown ceramic discs was used at spot 1. Here too, the transient counts data vary similarly to the data of the SMT100, showing the irrigation and drying cycles. However, since the pores of the brown ceramic discs were much smaller than those of the white ceramic discs, they retain the stored water more strongly, and, as a result, the drying cycles in particular show up less pronounced. Here, the measured values increased to a maximum of 3600 counts after 800 h.

These measurements show in particular that the installation location of the sensor is significant for the obtained results. Even small differences in the distance to the irrigation unit among the individual sensors can cause significant differences in the obtained results. Basic irrigation or drying trends, however, were properly detected by all the sensors at all the installation locations in this trial.

- Spinach research field:

The subsequent experiments in the spinach field at LVG were similar to those in the tomato field, but, this time, sensors with brown ceramics were installed at both spots and irrigation was carried out more regularly (five irrigation events). Furthermore, the measuring domain of the TEROS 21 was reduced to a range of 0 to −23 kPa.

The temperature in the soil started at approximately 18 °C on 7 October and steadily decreased to approximately 8 °C on 30 December. Day and night fluctuations in the range of ±1 °C during the whole measuring process were reflected equally by all the sensors.

In the spinach field experiments, a total of five irrigation events were recorded by all the sensors at both spots: after 35 h, 220 h, 730 h, 970 h, and 1500 h. If irrigation 3 at 730 h is neglected, all the irrigations that occurred after similar readings were obtained for the respective sensor at both spots with non-significant variations; the data are summarized in Table 3:

The differences between spot 2 and 1 were negligible for the sensor with white ceramic discs, the TEROS 21, and the SMT100. Only the sensors with brown ceramic discs showed a significant difference of 276 counts on average between the two spots. This could be due to the narrow pore size distribution and small average pore size in this ceramic. Due to the small pores, the ceramic might not be able to establish a hydraulic equilibrium with the different particle size ranges in the soil and thus may exhibit a significantly higher dependence on the correct positioning during installation.

The sensor with white ceramic discs provided promising results similar to the TEROS 21 in determining the plant-available water in the soil. Compared to the SMT100, the transient data provided by these sensors show similar variations over time, with high accuracy and little scatter.

#### 3.3.4. Rhineland Palatinate High Forest: Hermeskeil

**Figure 8:**
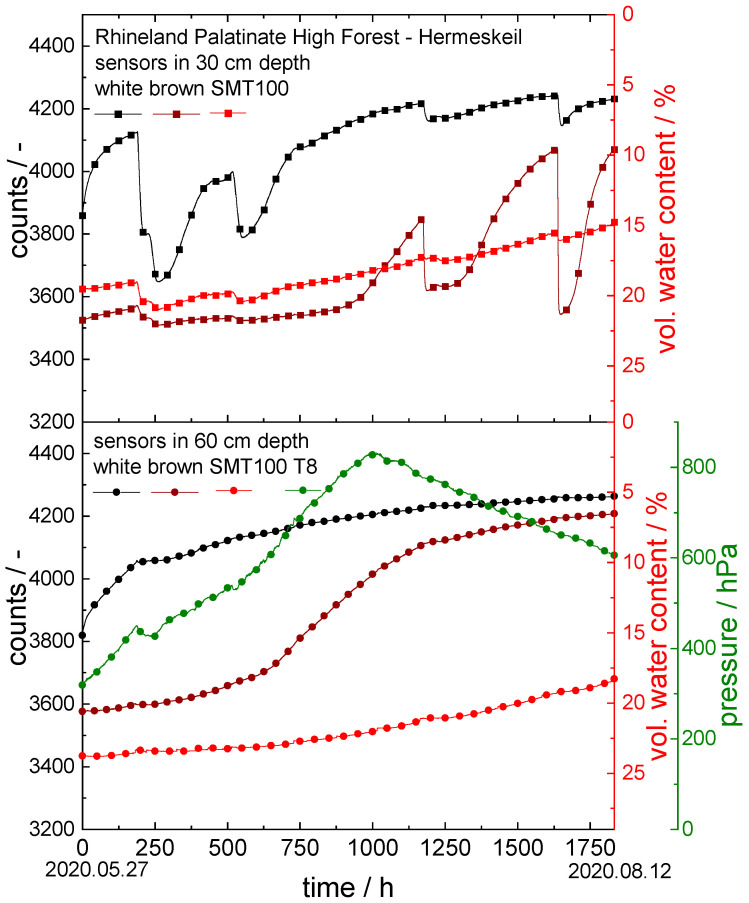
Drying experiment in forest soil (Rhineland Palatinate High Forest: Hermeskeil); counts are plotted vs. time in hours for the sensors with the ceramic discs; vol. water content vs. time for the SMT100 and soil water potential vs. time for the T8 tensiometer. Time = 0 h corresponds to the date 27 May 2020, and time = 1833 h to 12 August 2020.

For the forest field experiments, sensors mounted with brown and white ceramics and a dielectric sensor (SMT100) were installed at a measuring station at Hermeskeil in the Rhineland Palatinate High Forest in cooperation with the Landesforsten Rhineland-Pfalz forestry administration. At the measuring station, a tensiometer (T8) was already installed in a depth of 60 cm. The six additional sensors were mounted in a depth of 30 cm and 60 cm. The measuring period examined was between 27 May 2020 and 12 August 2020. The results of the soil moisture measurements are shown in Figure 8. In this case, irrigation took place only through natural precipitation.

All three sensors used at a depth of 30 cm registered the precipitation events after 210, 550, 1200, and 1650 h. According to the measurements, however, the forest soil dried out over the whole measuring period. The measured values of the sensor with white ceramic discs increased over the measuring period from 3860 to 4230 counts and those with the brown ceramic discs from 3530 to 4070 counts. At the same time, the water content according to the SMT100 decreased from 19.5 to 14.8 vol.%.

The data recorded with the sensors at a depth of 60 cm are similar to those at a depth of 30 cm. The difference, however, is that the precipitation events are not registered by the sensors at a depth of 60 cm. Only for the first event after 210 h did the sensor with white ceramic discs, as well as the T8 tensiometer, show a slight drop in the measured values. Overall, the long-term drying out of the soil became even clearer at a depth of 60 cm: here, the count values detected by the sensor with white ceramic discs increased from 3820 to 4260 counts and those registered by the sensor with the brown ceramic discs increased from 3580 to 4210 counts. At the same time, the volumetric water content according to the SMT100 decreased from 23.8 to 18.4 vol.%. The T8 tensiometer registered an increase in the soil water potential to the degree of drying out completely after 1000 h, reaching the maximum reading of 825 hPa. This is followed by the typical drying-out behavior of such a kind of tensiometer itself, in which the measured values decrease constantly but not in relation to a change in soil humidity.

This experiment revealed a great disadvantage of the commercial tensiometer, which was no longer functional above a certain soil water potential and required increased maintenance. Especially in remote areas, our sensor offers a valuable alternative since it does not require any service and can still measure far beyond the soil water potential limit specified by the classical tensiometers (here, 825 hPa).

## 4. Discussion

To make irrigation efficient and resource-saving, the water available to plants in the soil should be determined reliably and accurately. With this goal in mind, we developed a novel sensor and presented it for the first time in ref. [34].

In order to retrieve the soil-available water for plants, this sensor detects the resonance frequency of an electrical circuit, which includes two ceramic discs as dielectric layers in a capacitor layout. The resonance frequency depends on the water content in these discs, i.e., the amount of water absorbed from the surrounding soil, and is subsequently converted into so-called counts as an output signal of the sensor system. However, the counts are only raw data, which should be converted into the soil water potential since this parameter is generally used to characterize the water available for plants.

**Figure 9:**
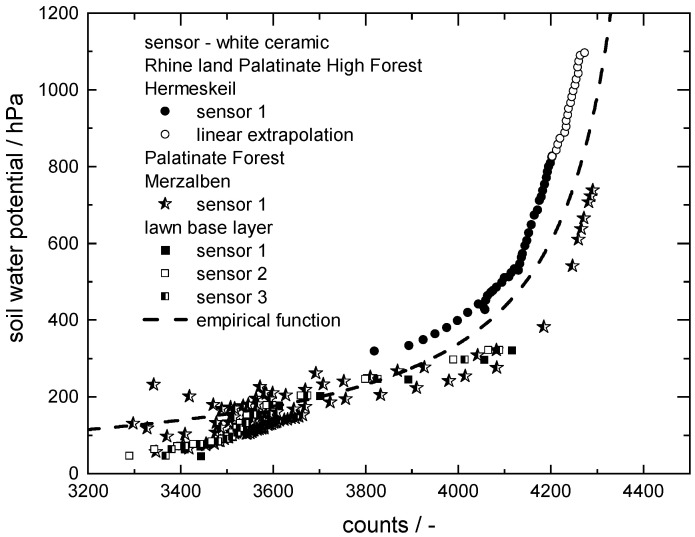
Correlation of soil water potential data (T8) and count values from the sensor with white ceramic discs recorded during drying experiments in model soil lawn base layer [34], as well as forest soil (Palatinate Forest: Merzalben [34] and Rhineland Palatinate High Forest: Hermeskeil).

To achieve this, the soil water potential obtained from a commercial T8 tensiometer was plotted as a function of the count values recorded with the novel sensor mounted with white ceramic discs during forest soil experiments in Hermeskeil and Merzalben [34], as well as in laboratory experiments in a lawn base layer [1]. The corresponding data are shown in Figure 9. During the experiments in Hermeskeil, the upper measurement limit of 850 hPa of the T8 tensiometer was exceeded, but the ceramic disc sensor still recorded data during further drying of the soil. A linear correlation was assumed to extrapolate water potential data corresponding to these count values (shown as open symbols in Figure 9). An empirical function was fitted to the whole set of data points:

(2) $$\mathrm{SWP}=f(\mathrm{counts})=\frac{1}{a+b\cdot counts^{c}}\;a=0.1\;b=-0.01\;c=0.274$$

Based on this function, the count values from the new ceramic sensor can be converted into soil water potential data with a reasonable accuracy ±100 hPa; no additional knowledge regarding the soil composition is required.

In the wet regime around 3400 counts, the measured data show irregular scatter. This could be due to the delayed data logging of the T8 tensiometer, which occurs during abrupt changes in soil moisture, such as heavy rain. Our sensor does not exhibit this delay. As a result, measured count values are assigned to soil water potential values that are too high. In addition, hysteresis in the irrigation and drying process occurs with tensiometers, such as T8 [40]. For this reason, fluctuations of ±50 hPa in the soil water potential up to 3800 counts are present in the correlation.

Particularly in the dry regime >4000 counts, the soil water potential values obtained at a given count value in the Merzalben soil are systematically higher than those recorded in the Hermeskeil soil. This could be due to the different particle size distributions in these soils. Since the soil in Hermeskeil includes a larger fraction of small particles, the water is bound more strongly there, especially in the dry regime. The sensor, including the white ceramic discs with a wide pore size distribution, was explicitly designed to form a hydraulic equilibrium with all the soils. However, there are apparently still systematic differences in the range of ±100 hPa in the measured data when the particle size distribution in the soil differs greatly. The brown ceramic has a narrow pore size distribution and, for this reason, is not suitable for use in different soils aiming at a soil independent correlation between the SWP and measured counts.

With our novel sensor and the empirical model proposed in Equation (2), it is now possible to accurately characterize the soil water potential from very wet to very dry conditions up to 1200 hPa independent of the soil type, with an accuracy ±100 hPa. Further, the reproducibility of the SWP determination in a single given soil is ±15 hPa.

## 5. Conclusions

In ref. [34] a novel sensor for soil moisture characterization was presented. This device includes a printed circuit board with a tailored electronic layout and two porous ceramic discs mounted on it. Dielectric measurements are used to determine the amount of water absorbed by the discs, which is considered to be related to the soil water potential. The porous ceramic discs were fabricated using an innovative processing route employing so-called capillary suspensions as a pre-cursor [36,38], allowing for tailoring a wide pore size distribution, very high porosity, and sufficient mechanical strength. The novel sensor is a highly sensitive and robust device with a wide measurement range, as confirmed here in a large number of field trials in direct comparison with competing products.

In this paper, we were able to show that the new sensor can be used in a variety of different soils with a diversity of crops and a wide range of conditions. The new sensor provides accurate and reliable results; in particular, the experiments with controlled irrigation have shown that the sensor provides data with high accuracy and low scatter. It is thus a valuable tool, suited for optimizing irrigation in agriculture.

Based on a comparative study including three very different soil types, a universal empirical relationship between the output signal of the new sensor and the soil water potential, as obtained using a T8 tensiometer, was established, which allows for the determination of the soil water potential without knowledge regarding the soil composition.

## Figures and Tables

**Table 1:** Commercially available sensor systems. All information according to the respective manufacturer.

System Name	Manufacturer	Measuring Range	Approx. Price	Source
TEROS 21 (former MPS-6)	METER Group, Inc. (Pullman, WA, USA) (former Decagon Devices, Inc.)	90 to ∞ hPa	250 €	[26]
Watermark (granular matrix)	Irrometer Company, Inc. (Riverside, CA, USA)	0 to 2000 hPa	70 €	[27]
EQ3 Equitensiometer	Delta-T Devices Ltd. (Burwell, Cambridge, UK)	0 to 10,000 hPa	800 €	[28]
Tensiomark (0–7)	EcoTech Umwelt-Meßsysteme GmbH (Bonn, Germany)	1 to 10,000,000 hPa	650 €	[29]
Full Range (Polymer-) Tensiometer	Wageningen University (Wageningen, The Netherlands)	0 to 15,000 hPa	1000 €	[30,31,32]

**Table 2:** Data of ceramic characterization of brown and white kieselguhr and TEROS 21 ceramic.

Ceramic Type	Diameter/mm	Height/mm	x_10,3_/μm	x_50,3_/μm	x_90,3_/μm	ε_open_/%	σ/MPa
brown ceramic	43.0 ± 0.2	5.0 ± 0.2	16	41	72	59 ± 3	12 ± 3
white ceramic	43.0 ± 0.2	5.0 ± 0.2	44	101	159	69 ± 3	6 ± 1
TEROS 21	31.5 ± 0.5	2.9 ± 0.1	45	93	148	62 ± 2	6 ± 1

**Table 3:** Data for the irrigation events at spots 2 and 1.

Spot	White/Counts	TEROS 21/kPa	SMT100/%	Brown/Counts
2	3484 ± 10	11.7 ± 0.5	16.5 ± 2.8	3292 ± 35
1	3406 ± 25	11.9 ± 0.6	16.5 ± 2.8	3567 ± 10

## Data Availability

Not applicable.
